# Argentum Nitricum

**Published:** 1887-06

**Authors:** J. T. Kent

**Affiliations:** St. Louis


### ARGENTUM NITRICUM.

### A Lecture by J. T. Kent, M. D., St. Louis.

Nitrate of silver is a wonder, like all other medicines after careful study and use. It is an antisycotic, and cures its own kind of bleeding warts and excrescences. It often acts wonderfully in small-pox and in the complaints that follow vaccination. The complaints are all made better in the open air, even in the cold winds. He craves the cold wind blowing in his face and lungs. This has been a strong symptom in the last stages of consumption, catarrhal chest affections, and asthma. The mucous membranes all suffer with inflammation and ulceration, especially the throat and os-uteri. The skin takes on a bluish colored erysipelas, and may ulcerate. Pressure causes quick ulceration—as in bed-sores—and all inflamed and ulcerated surfaces have the sensation of being full of sticks (Nitr. ac., Hep.). Inflamed surfaces feeling like sticks, as in Natr. mur., Alumina. It competes with Puls., Iodine, Sabin., in craving the cool open air.

Mucous surfaces bleed easily, and the hemorrhage is dark, even black. Coition is followed by a discharge of blood from the spongy, sycotic excrescences growing from the os and sides of the vagina. This wonderful medicine produces the "skimmed milk" countenance, and the cachectic aspect of sycosis, even the greasy skin of Thuj., is reproduced with its shining horror.

Suppressed gonorrhœa in women, when the right ovary becomes painful and enlarged, the symptoms often fully correspond to Arg. n. The greasy, shining face is now characteristic of Arg. n., Natr. m., and Thuj. It is not astonishing to observe that Natr. m. is its antidote, as well as its complement. This medicine causes emaciation from below upward, unlike its complement, Natr. m., which causes emaciation from above downward, commencing about the neck and arms (Lycop.).

It has a violent craving for sugar, which often brings on a diarrhœa of grass-green stools. Cholera infantum with green stools in a baby, that has been nursed by a mother that eats candy, commences to emaciate about the legs, and finally takes on the look of a little old person. Can any student of materia medica fail to see the analogue of Natr. m. in this medicine? The marasmus of the Natr. m. cases is much like this; the face is old, the infant is violently thirsty, and while it eats all it can get ravenously, it continues to lose flesh. Throbbing all over the body, the carotids thump, and the headaches beat like hammers.

Fulgurating pains, paralytic weakness, and great trembling. Marked chilliness, especially in a warm bed. Perspiration and chilliness. He must go into the open air. Restless, must keep in continual motion. Extreme nervousness, and prostration, fainting, excitement, and frenzy; hysteria and epilepsy; convulsions; paralysis of lower extremities; staggering, jerking in the limbs during sleep; numbness in the legs; formication in the skin; legs feel as if made of wood (Ars.). He staggers on closing the eyes; hands numb and tingle; loss of sensation in the fingers. With all these evidences of spinal symptoms, we find great palpitation in high degree on the slightest mental emotion.

Next among the generalities and characteristics is the marked flatulence that attends the stomach and bowel symptoms.

Vomiting and purging in the same common effort is a symptom of great importance.

In this, and in the peculiar cough, we have an aggravation in relation to time. Just before midnight. When called to a dry cough that comes every night before midnight, and is violent and convulsive, coughing and gagging, belching, spitting up blood mixed with mucus, and caused by a fit of anger, laughing, or sudden exertion—a suffocating cough—Arg. n. should not be forgotten.

Irritating dry cough.

Chronic hoarseness of singers; loss of voice.

It is common for the rawness of the larynx to alternate with uterine troubles. When the one is better the other is worse. I once had a patient come to me for her throat. She had taken her uterus to a gynæcologist. I soon ascertained that she had withheld some of the symptoms, and a confession soon followed. Of course, the gynæcologist was deprived of occupation in the case.

Arg. n. has removed pterygium, so has Zinc, but he who prescribes either of these to remove pterygium knows not the spirit of Homœopathy, and he who thinks he has proved that Carroll Dunham has made a mistake because Zinc will not cure this growth again, demonstrates his own lack of knowledge of the homœpathic healing art. Arg. n. causes the very worst form of conjunctivitis, with granular lids, burning, sticking, splinter sensation; chemosis with purulent discharge.

The mental symptoms are no less wonderful and peculiar. The controversy between will and reason is as strongly represented in this medicine as in Anac. It belongs to Arg. n., Anac., Op., Cannab. ind., Thuj.

He was utterly bereft of all power of will.

Against whatever was proposed he had the queerest objection. Fears that passing a certain corner he will drop down and create a sensation.

He frequently settles upon the day of his death, like Acon. The sense of his weakness comes upon him, and although he looks perfectly well, he feels that he will give out if he undertakes to work. Feels really incompetent to undertake either mental or physical exertion. Both, or either, aggravate his complaints.

This marked feeling of inability often comes on in sycotic affections of the mind. I have seen it many times, after suppressed gonorrhœa, and it disappears on the reappearance of the discharge. I have seen the discharge reappear in a milky flow, or thin and watery, as late as twelve years after its suppression. I am perfectly conversant with the usual doctrines held, and how skeptically this remark will be considered, but it does not change me back to the skepticism of my old belief, when I thought I was acquainted with pathology and disease. I hope my folly will cease with my youth.

This drug produces and cures the most marked hypochondriasis, and is full of illusions and imaginations, which he makes a great effort to reason down ; but these fancies are fixed, and the struggle is useless.

Fears, loss of memory, sadness and suicidal. When he sees a deep pit or stands on a high place he feels that he must jump in. Inclined to take his life, but lacks the courage. He is full of deception and cowardice, and he knows it, and makes a great effort to keep others from finding it out. He has the most extreme anxiety and fear when alone (like Phos.). He always fears something is going to happen. He will not take a high room in a hotel at night, fearing he might get up in his sleep and jump out of the window. He fears he may be seized with an impulse he cannot control. There is a strong resemblance to Anac., but the cursing is not in Arg. n., nor the marked lack of religious feeling in religious people. It strangely resembles Thuj. in the symptoms of fixed ideas, although Anac. also has it.

The mental agitation is peculiar, and shows wonderful weakness. When preparing for church, or theatre, or to meet an engagement, he becomes fidgety, trembles, and gets diarrhœa. Fear or excitement brings on diarrhœa. (Gels.)

There are many headaches, congestions, constrictions, boring, digging, cutting pains. Most pains are ameliorated by a close-fitting bandage.

